# PGC-1α activation boosts exercise-dependent cellular response in the skeletal muscle

**DOI:** 10.1007/s13105-024-01006-1

**Published:** 2024-01-23

**Authors:** Soroosh Mozaffaritabar, Erika Koltai, Lei Zhou, Zoltan Bori, Attila Kolonics, Sylwester Kujach, Yaodong Gu, Atsuko Koike, Anita Boros, Zsolt Radák

**Affiliations:** 1 Research Institute of Molecular Exercise Science, Hungarian University of Sports Science, 1123 Budapest, Hungary; 2 Department of Neurophysiology, Neuropsychology and Neuroinformatics, Faculty of Health Sciences, Medical University of Gdansk, 80-210 Gdansk, Poland; 3 Faculty of Sports Science, Ningbo University, Ningbo, 315211 China; 4 Waseda Institute for Sport Sciences, Waseda University, Saitama, 359-1192 Japan

**Keywords:** Skeletal muscle, PGC-1α overexpression, Exercise, Mitochondrial function, Lipid Metabolism

## Abstract

The role of Peroxisome proliferator-activated receptor-gamma coactivator alpha (PGC-1α) in fat metabolism is not well known. In this study, we compared the mechanisms of muscle-specific PGC-1α overexpression and exercise-related adaptation-dependent fat metabolism. PGC-1α trained (PGC-1α Ex) and wild-trained (wt-ex) mice were trained for 10 weeks, five times a week at 30 min per day with 60 percent of their maximal running capacity. The PGC-1α overexpressed animals exhibited higher levels of Fibronectin type III domain-containing protein 5 (FNDC5), 5' adenosine monophosphate-activated protein kinase alpha (AMPK-α), the mammalian target of rapamycin (mTOR), Sirtuin 1 (SIRT1), Lon protease homolog 1 (LONP1), citrate synthase (CS), succinate dehydrogenase complex flavoprotein subunit A (SDHA), Mitofusin-1 (Mfn1), endothelial nitric oxide synthase (eNOS), Hormone-sensitive lipase (HSL), adipose triglyceride lipase (ATGL), G protein-coupled receptor 41 (GPR41), and Phosphatidylcholine Cytidylyltransferase 2 (PCYT2), and lower levels of Sirtuin 3 (SIRT3) compared to wild-type animals. Exercise training increased the protein content levels of SIRT1, HSL, and ATGL in both the wt-ex and PGC-1α trained groups. PGC-1α has a complex role in cellular signaling, including the upregulation of lipid metabolism-associated proteins. Our data reveals that although exercise training mimics the effects of PGC-1α overexpression, it incorporates some PGC-1α-independent adaptive mechanisms in fat uptake and cell signaling.

## Introduction

Skeletal muscle serving as a main organ for contraction and locomotion also plays an important role in fatty acid oxidation (FAO) [1]. During rest and moderate exercise intensity, lipids cover most of the energy needed to maintain homeostasis and physical activity [2]. Even in male subjects with a normal body mass index, endogenous fat stores are huge compared to carbohydrates, and the total energy storage can be as high as 140,000 kcal [3]. Interestingly, at the same level of physical activity, there is a reduction of FAO in obese people leading to further accumulation of triacylglycerol in subcutaneous and deep visceral adipose tissue stored fat [4]. Skeletal muscle, which is the greatest organ stores a significant amount (2–10 mmol/kg wet wt) of intramuscular triacylglycerol, which could be readily used during muscle contractions, since intramyocellular lipid droplets are located around the mitochondrial network [3]. The intensity of exercise is one of the major factors which influence the contribution of different energy sources to cover the energy need of exercise [5]. At low-intensity exercise the major source of energy is gained from free fatty acids (FFA), while during moderate intensity (40–60% of VO2max), approximately half of the fat-based energy is served by FFA and intramuscular triacylglycerol. At higher intensity than 70% of VO2 max, the use of fat as an energy source significantly drops and will be just a minor contributor of energy production [3].

When the molecular mechanism of adaptive thermogenesis was studied, PGC-1α was cloned and found to increase the cellular content of mitochondrial DNA and link nuclear receptors to the transcriptional program of adaptive thermogenesis [6]. Later on, it was also found that PGC-1α regulates a wide range of cellular signaling, and it turned out that the metabolic sensor AMPK-α is one of the main upstream regulators of PGC-1α, which positioned this co-activator as a key regulator of cellular metabolism as well. Moreover, it has been shown that PGC-1α is involved in fat metabolism [7], however, its role is not completely understood. In this study, we compared the possible mechanisms behind PGC-1α overexpression and exercise adaptation-related fat metabolisms.

## Material and methods

### Animals

We randomly allocated 40 male C57BL/6-Tg(Ckm-Ppargc1a)31Brsp/J mice, all of which were 10 months old, into four distinct groups: Wild-type Control (wt-C) and PGC-1α control (PGC-1α-C), each consisting of 11 animals (n = 11); and exercise groups wild-type Exercise (wt-Ex) and PGC-1α Exercise (PGC-1α-Ex), with 9 animals in each group (n = 9). The animals were kept in the animal house of the Research Center for Molecular Exercise Science at the University of Physical Education of Hungary. They were subjected to a 12/12-h day-night cycle and had unrestricted access to standard laboratory food and water. The study was ethically approved by the National Animal Research Ethical Committee of Hungary (Approval No. PE/EA/62–2/2021).

### Training protocol

After familiarizing the mice in the training groups with the treadmill, we used a fatigue endurance test to practically measure their maximal running capacity, a key metric for calculating the animals average running speed. According to the data obtained from the endurance test, we started the training at a 60 percent of the animals' maximal running capacity and gradually increased the exercise intensity each week. The training continued for 10 weeks, with five days of 30-min training sessions per week. Following the completion of the exercise intervention, animals were sacrificed in a compassionate and ethical manner to minimizes any potential pain, suffering, or distress for removing their quadriceps muscles. The muscles we took out were quickly put into liquid nitrogen to keep them in good condition. Later, we stored them in a freezer at -80 °C. This careful preservation method makes sure the muscle samples stay in the best condition for further, detailed analysis.

### Tissue homogenization

We separated 50–80 g of the quadriceps muscle in the same way for all the samples and homogenized them by using a lysis buffer containing 137 mM NaCl, 20 mM Tris–HCl pH 8.0, 1% Nonidet P-40, 10% glycerol plus protease SIGMAFAST™ Protease Inhibitor Tablets (S8820-20TAB) and PhosSTOP™ phosphatase (4,906,845,001) inhibitor tablets in order to prevent degradation. The muscle tissues were placed in this lysis buffer within an ice container, followed by homogenization using an Ultra Turrax homogenizer (IKA, Staufen im Breisgau, Germany). After having homogenized tissues, we measured protein content with the Bradford assay technique for determining their protein content to make all samples have equal amounts of protein for loading into polyacrylamide (SDS-PAGE) gels for western blots.

### Western blots

After having proper samples for loading in 8–12% SDS-PAGE, we loaded 4–8 μl of samples inside the gels to perform electrophoresis. Following the completion of the electrophoresis process, we transferred the proteins from the gels to PVDF membranes using the Trans-Blot® SD Semi-Dry Electrophoretic Transfer Cell (1,703,940). Then by using a 5% Milk Tris-buffered saline-Tween-20 (TBST) solution, we blocked the membranes for one hour at room temperature. The incubation step of the Target-specific antibody took place at night-time in the 4 °C fridge. The primary antibodies used for incubation include: HSL rabbit (1:1000, cell signaling 18381S), ATGL rabbit (1:1000, cell signaling 2439 S), mTOR rabbit (1:1000, cell signaling 2983S), Sirt3 rabbit (1:1000, cell signaling 2627), PCYT2 rabbit (1:1000, Thermofisher PA5-90,366), AMPK-α rabbit (1:1000, cell signaling 2532), eNOS mouse (1:1000, abcam ab76198), Phospho-eNOS (Ser1177) rabbit (1:1000, cell signaling (9571), Sirt1 mouse (1:1000, abcam, ab110304), LONP1 mouse (1:3000, Proteintech − 66,043–1-Ig), SDHA rabbit (1:3000, SantaCruz—sc-98253), CS rabbit (1:1000, abcam,ab96600), PGC-1α rabbit (1:3000, Novusbio, NBP1-04676), Fis1 rabbit (1:1000, SantaCruz, sc98900), Mfn1 rabbit (1:1000, SantaCruz, sc50330), FNDC5 rabbit (1:1000, abcam, ab174833), GPR41 rabbit (1:1000, Thermofisher, PA5-75,521), GPR43 rabit (1:500, Thermofisher, PA5-111,780), nNOS mouse (1:1000, BD Transduction Laboratories, 610,309), NAMPT/Visfatin rabit (1:1000,abcam, ab45890), Glyceraldehyde 3-phosphate dehydrogenase (GAPDH) mouse (1:40,000;Sigma-Aldrich, 9001–50-7), α-Tubulin mouse (1:20,000, Sigma-Aldrich,T6199). On the following day, the incubated membranes went through a washing process with TBST buffer 4 times 8 min at room temperature then we performed the second antibody incubation process in room temperature for one hour according to the host species of primary antibody the anti-rabbit, anti-mouse IgG HRP-conjugated secondary antibodies (Jackson Immunoresearch) were used which were diluted 1:10,000 in 5% milk TBST solution. Then the membranes were washed again with TBST buffer 4 times 8 min at room temperature. The washed membranes were incubated for one minute in chemiluminescent reagent SuperSignal™ West Pico PLUS Chemiluminescent Substrate REF-34580). We used AZURE 400 Visible Fluorescent Imager (AZI400-01) for detecting specific protein bands on the membranes by applying Wide Dynamic Range cumulative imaging mode. After obtaining band spots on the membranes we used ImageJ software version 1.53t for quantifying them. The values were normalised by housekeeping proteins which in our study were GAPDH and α-Tubulin. Also, the Phosphorylation ratio was calculated by using the same PVDF membrane after stripping the phosphorylated form of the proteins and incubating the same membrane in the total protein antibodies.

### Statistical analysis

The statistical analyses were performed using GraphPad Prism version 9.1.0 software. All data are expressed as the mean and standard deviation (SD), or standard error of mean (SEM). The level of significance was set as p < 0.05 for all of the analyses. Additionally, a two-way analysis of variance (ANOVA) was used to investigate the significance of differences between groups and conditions. Significant main effects were further analyzed using Fisher's post hoc test.

## Results

### The effects of PGC-1α overexpression on running distance

**Fig. 1:**
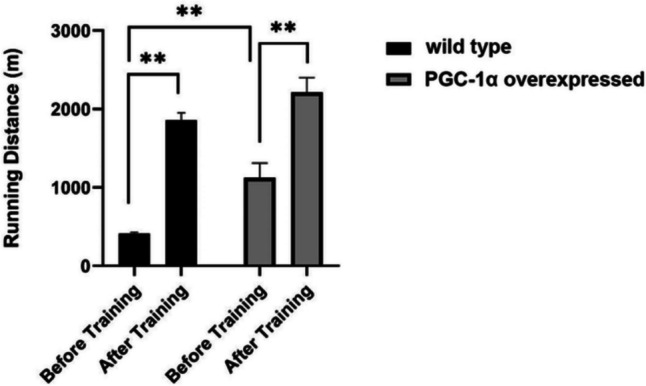
Comparison of average running distance to exhaustion before and after exercise training between the wt-Ex and PGC-1α-Ex groups. The results are presented as mean ± SEM, with statistical significance determined using two-way ANOVA and Fisher's LSD test (*p < 0.05, **p < 0.01)

The comparison of means of running distances to exhaustion revealed a significant difference between wild type (wt-Ex) and PGC-1α overexpressed (PGC-1α-Ex) animal groups at the baseline as well as before and after the exercise training. This important finding shows that when PGC-1α is overexpressed in skeletal muscle, it can make endurance activities better and help the body to enhanced fatigue resistance (Fig. 1).

### The effects of PGC-1α overexpression and exercise training on mitochondrial and oxidative stress marker levels

**Fig. 2:**
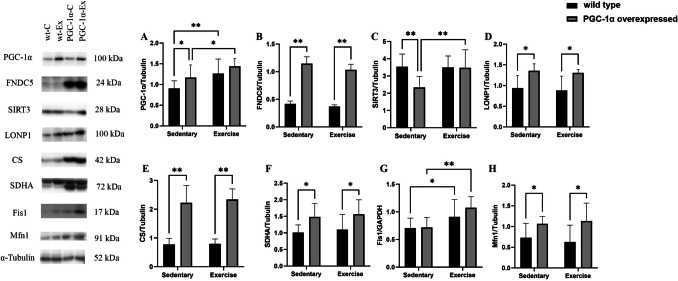
The effects of PGC-1α overexpression and exercise training on mitochondrial and oxidative stress marker levels proteins in the mice quadriceps muscle. Sedentary (n = 11) groups, Exercise groups (n = 9). all Western blot analysis was conducted on selected bands, and their quantification was normalized to GAPDH and α-Tubulin. The results are presented as mean ± SD, with statistical significance determined using two-way ANOVA and Fisher's LSD test (*p < 0.05, **p < 0.01)

The PGC-1α overexpressed animals (PGC-1α-C) had higher levels of PGC-1α (A), FNDC5 (B), LONP1 (D), CS (E), SDHA (F), Mfn1 (G), and lower levels of SIRT3 (C) compared to wild-type animals (wt-C). Exercise training increased the levels of the protein content of PGC-1α (A), Fis1 (G) in group whereas PGC-1α (A), SIRT3 (C), and Fis1 (G) levels increase in the PGC-1α-Ex group (Fig. 2).

### The effects of PGC-1α overexpression and exercise training on metabolic and adaptive capacity-related markers

**Fig. 3:**
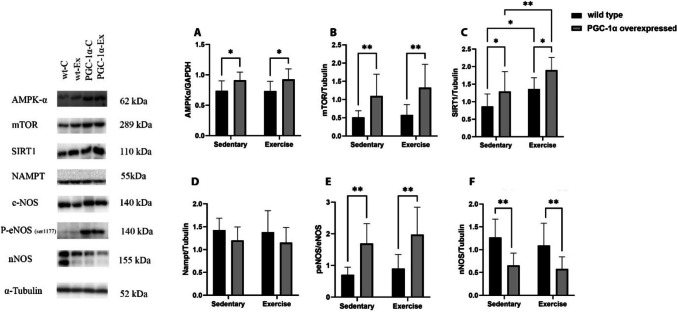
The effects of PGC-1α overexpression and exercise training on metabolic and adaptive capacity related protein levels in the mice quadriceps muscle. In comparison to wild-type animals, PGC-1α overexpressed animals (PGC-1α-C) showed a significant increase in AMPK-α (**A**), mTOR (**B**), SIRT1 (**C**) and peNOS/eNOS (**E**) whereas significant decrease has been observed only in nNOS (**F**). Also, SIRT1 (C) protein levels increased in both exercised groups (wt-Ex and PGC-1-Ex), where the increase was more pronounced among PGC-1-Ex group. Sedentary (n = 11) groups, Exercise groups (n = 9). Western blot analysis was conducted on selected bands, and their quantification was normalized to GAPDH and α-Tubulin. The results are presented as mean ± SD, with statistical significance determined using two-way ANOVA and Fisher's LSD test (*p < 0.05, **p < 0.01)

The PGC-1α overexpressed animals (PGC-1α-C) had higher levels of AMPK-α (A), mTOR (B), SIRT1 (C), peNOS/eNOS (E), and decreased levels of nNOS (F) compared to wild-type animals (wt-C). Moreover, exercise training increased the levels of the SIRT1 (C) protein content in the wt-Ex as well as PGC-1α-Ex group (Fig. 3).

### The effects of PGC-1α overexpression and exercise training on lipid metabolism markers

**Fig. 4:**
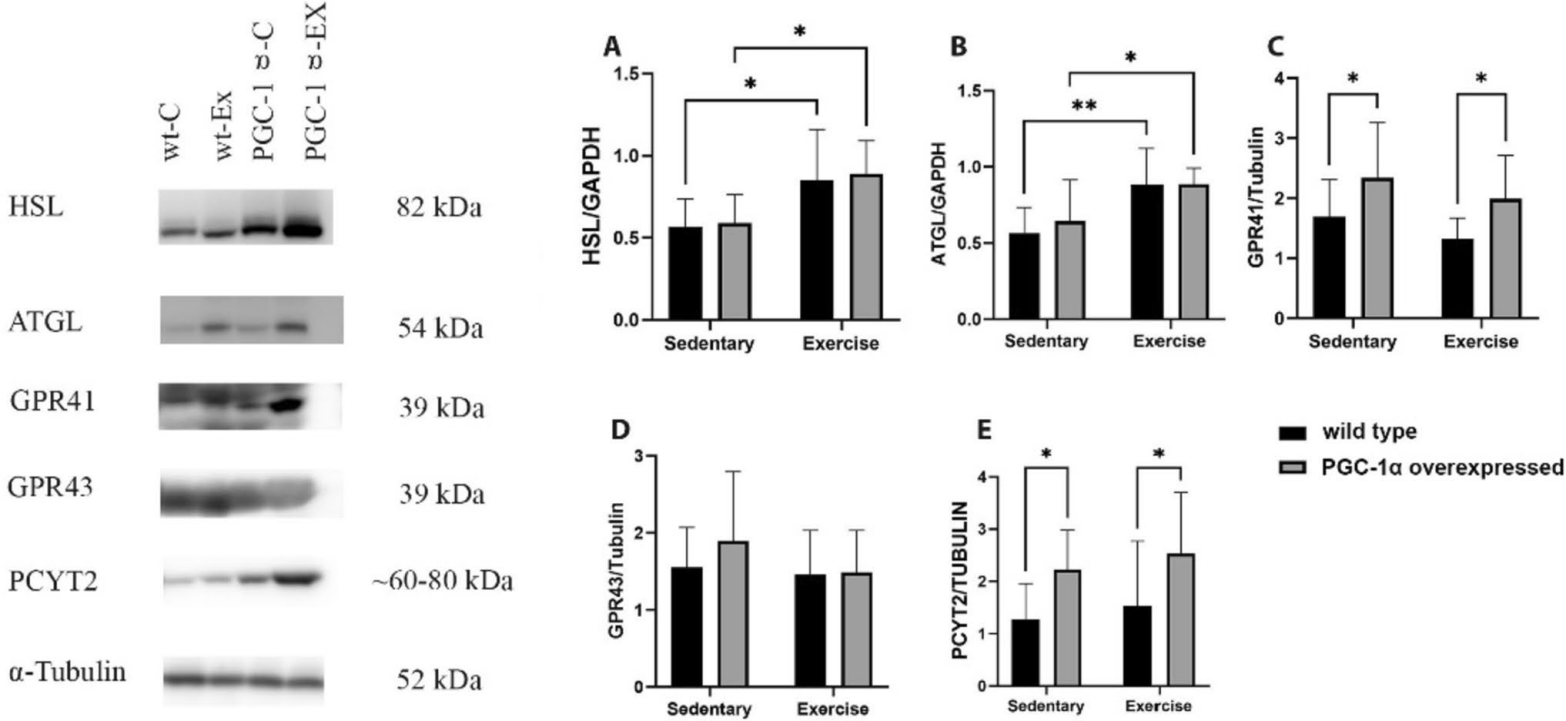
The effects of PGC-1α overexpression and exercise training on the levels of lipid metabolism related proteins in the mice quadriceps muscle. PGC-1α overexpressed animals (PGC-1α-C) showed significant increase of GPR41 (**D**), and PCYT2 (**E**) when compared to the wild-type animals (wt-C). Additionally, exercise training significantly increased the levels of HSL (A) and ATGL (B) key lipases involved in triglyceride breakdown among both groups (wt-Ex and PGC-1α-Ex). Sedentary (n = 11) groups, Exercise groups (n = 9). Western blot analysis was conducted on selected bands, and their quantification was normalized to GAPDH and α-Tubulin. The results are presented as mean ± SD, with statistical significance determined using two-way ANOVA and Fisher's LSD test (*p < 0.05, **p < 0.01)

The PGC-1α overexpressed animals (PGC-1α-C) exhibited higher levels of GPR41 (C), and PCYT2 (E) compared to wild-type animals (wt-C), while exercise training increased HSL (A), and ATGL (B) levels in the wild-type (wt-Ex) group as well as PGC-1α overexpressed animals (PGC-1α-Ex) (Fig. 4).

## Discussion

It has been shown that PGC-1α overexpression leads to increased lipolysis via upregulation of the pentose pathway [7]. However, here we show that the mobilization and uptake of fatty acids also influenced PGC-1α. PGC-1α overexpression in skeletal muscle resulted in elevated levels of FNDC5, which releases irisin into circulation and tissues take it up through αV class integrin receptors [8]. The irisin-integrin axis could lead to enhanced lipolysis due to activation of HSL [9], a pivotal enzyme initiating the lipolysis process, is indeed confirmed in our study. Moreover, irisin can activate the ATGL enzyme [9] which promotes lipolysis. Indeed, activation of ATGL leads to the release of fatty acids from triacylglycerol (TG) [10] and our data revealed that PGC-1α overexpression causes a significant and exercise-like increase in ATGL levels in the quadriceps muscle.

GPR41 is a fatty acid receptor specialized for up-taking Short-chain fatty acid (SCFA), which is mainly produced by the fermentation of dietary fibers by gut bacteria [11]. Here we show that PGC-1α overexpression increases the levels of GPR41 compared to wild-type animals, indicating enhanced uptake of SCFA into skeletal muscles of PGC-1α overexpressed mice in PGC-1α-C and PGC-1α-Ex. Phosphoethanolamine cytidylyltransferase (PCYT2) is a crucial enzyme in lipid biosynthesis [12]. It functions as an enzyme that catalyzes the formation of CDP-ethanolamine from CTP and phosphoethanolamine (PE) in the Kennedy pathway [13]. The importance of PCYT2 is emphasized through its involvement in the metabolic processes of phosphatidylethanolamine (PE), a crucial component found in different membranes, including those within cells and inner mitochondria [14, 15]. Moreover, this enzyme plays a role in maintaining optimal muscle function, particularly influencing mitochondrial bioenergetics, reactive oxygen species (ROS) production, and the lipid bilayer characteristics of myofiber membranes [14, 16]. Our recent study pointed out the importance of PCYT2, in muscle health, and PCYT2 depletion impaired mitochondrial function and muscle energetics [14]. Here we show that PCYT2 levels are elevated in the skeletal muscle of PGC-1α overexpressed mice, compared to wild-type animals, indicating enhanced PE synthesis as an adaptive response to increased mitochondrial biogenesis and PGC-1α dependent increase in exercise efficiency.

The upregulation of mitochondrial proteins by PGC-1α overexpression is an expected result, the increased levels of mitochondrial fission protein could be important to facilitate the generation and mobility of daughter mitochondria, to quality control, to remove damaged mitochondrial parts, mitochondrial energetics and signaling [17]. The PGC-1α overexpression is associated with increases in LONP1 levels, indicating improved mitochondrial protein quality control through removing damaged and malfunctioned mitochondrial proteins [18].

For cellular signaling, we assessed a number of proteins and in overexpressed animals we found increased AMPK-α, SIRT1, eNOS, and mTOR levels. AMPK-α is an upstream regulator of PGC-1α, but here we found that PGC-1α can also lead to activation of AMPK-α. PGC-1α can coactivate AMPK-α, leading to increased AMPK-α activity. This interaction occurs through multiple mechanisms. First, PGC-1α can directly bind to and activate AMPK-α, enhancing it kinase activity [19]. Second, PGC-1α promotes the expression of genes involved in fatty acid oxidation which are known targets of AMPK-α signalling [20].

Studies have demonstrated that PGC-1α can upregulate SIRT1 expression and its deacetylase activity [21]. PGC-1α binds to the promoter region of the SIRT1 gene and activates its transcription, thereby increasing the production of the SIRT1 protein. This interaction between PGC-1α and SIRT1 forms a positive feedback loop that regulates cellular energy homeostasis and mitochondrial function [21].

PGC-1α has been shown to have a positive feedback impact on endothelial function and nitric oxide (NO) production by eNOS. PGC-1α can directly coactivate transcription factors, such as peroxisome proliferator-activated receptor gamma, nuclear respiratory factor 1, and estrogen-related receptor alpha [6, 22]. These transcription factors bind to the promoter region of the eNOS gene and induce its expression. PGC-1α has been shown to stabilize eNOS mRNA, preventing degradation, prolonging half-life, and enhancing protein levels of eNOS [22]. PGC-1α can reduce the production of reactive oxygen species (ROS) and upregulate antioxidant enzymes. Excessive ROS can degrade eNOS protein, hence reducing ROS levels, PGC-1α helps to preserve eNOS protein and maintain its activity [23]. These mechanisms collectively contribute to the enhancement of eNOS levels by PGC-1α overexpression, leading to increased NO production and improved function.

mTOR exists in two complexes such as mTORC1 and mTORC2 [24], of relevance to PGC-1α, mTORC1 is particularly involved in the regulation of protein synthesis and cell growth [25]. It has already been shown that PGC-1α overexpression can increase mTOR activity by the following mechanism. PGC-1α can upregulate mitochondrial function and increase ATP production and nutrient availability are building blocks for mTORC1 activation [25].

In our investigation, we showed specific protein patterns, adaptive indicators, and metabolic pathways linked to the overexpression of PGC-1α and exercise through which PGC-1α influences and upregulates lipid metabolism and cell signaling in skeletal muscle. Notably, the pathways influenced by PGC-1α appear distinct from those associated with exercise alone. Specifically, based on expression of several critical markers, the muscle PGC-1α overexpression results in apparent activation of several metabolic processes even in the sedentary state, which is further amplified by exercise. This suggests that PGC-1α may exert its effects through unique mechanisms, providing valuable insights into the intricate interplay of molecular processes in muscle adaptation.

## Data Availability

All data and codes are available upon request. Western blot data are provided as supplementary material.
